# *Citrus* Pomace-Derived Plant Complexes Enhance Caco-2 Wound Closure In Vitro and Modulate Selected Probiotic Strains

**DOI:** 10.3390/antiox15091161

**Published:** 2026-09-11

**Authors:** Mariarosaria Ingegneri, Martina Imbesi, Souda Belaid, Marta Mangano, Maria Neve Ombra, Filomena Nazzaro, Antonella Smeriglio, Domenico Trombetta

**Affiliations:** 1 Department of Chemical, Biological, Pharmaceutical and Environmental Sciences, University of Messina, 98166 Messina, Italy; mariarosaria.ingegneri@unime.it (M.I.); martina.imbesi@studenti.unime.it (M.I.); souda.belaid@unime.it (S.B.); marta.mangano@unime.it (M.M.); domenico.trombetta@unime.it (D.T.); 2 Institute of Food Science, ISA-CNR, 83100 Avellino, Italy; nives.ombra@isa.cnr.it (M.N.O.); filomena.nazzaro@cnr.it (F.N.)

**Keywords:** *Citrus* pomace, plant complexes, gastrointestinal digestion, Caco-2 cells, epithelial wound closure, intestinal inflammation, oxidative stress, probiotic strains, cell-surface hydrophobicity, food by-products

## Abstract

*Citrus* processing by-products represent a sustainable source of bioactive plant complexes with potential applications in intestinal health. This study investigated the effects of standardized food-grade orange (OE) and lemon (LE) pomace extracts and their simulated gastrointestinal digestates (DIGs) on intestinal epithelial responses and selected probiotic strains. Caco-2 cells were used to assess cytotoxicity, epithelial wound closure, and, in differentiated monolayers challenged with lipopolysaccharide (LPS), extracellular levels of SOD2, catalase, Nrf2, IL-6, IL-8, TNF-α, and IL-1β. In parallel, the effects of OE, LE, and their corresponding DIGs on the growth and cell-surface hydrophobicity of four probiotic lactic acid bacteria were evaluated. OE and LE DIGs were non-cytotoxic and promoted epithelial wound closure in a concentration- and time-dependent manner. In LPS-challenged monolayers, both DIGs counteracted alterations in extracellular oxidative stress-related proteins and reduced pro-inflammatory cytokine levels without affecting cell viability. OE and LE also produced strain-dependent effects on probiotic growth and cell-surface hydrophobicity, which were modified by gastrointestinal digestion. Overall, these findings extend previous evidence on the intestinal bioactivity of *Citrus* pomace-derived plant complexes and support their further investigation as sustainable food-grade ingredients for gut health applications.

## 1. Introduction

The maintenance of intestinal homeostasis relies on a delicate balance among epithelial barrier integrity, redox homeostasis, immune responses, and the resident gut microbiota [1,2,3]. Disruption of this balance represents a key pathogenic event in inflammatory bowel disease (IBD), in which epithelial damage is not merely a consequence of inflammation but actively contributes to its perpetuation [4,5,6]. Increased intestinal permeability further facilitates the translocation of luminal antigens and microbial components, thereby sustaining immune activation and chronic inflammation [4,7]. Although the introduction of biologics and small-molecule drugs has significantly improved the clinical management of IBD, a substantial proportion of patients fail to achieve sustained remission or progressively lose their response to therapy, highlighting the need for complementary multitarget strategies capable of addressing the complex pathophysiology of the disease [8,9].

Among the mechanisms involved in the progression of intestinal damage, oxidative stress represents a major link between inflammation, barrier dysfunction, and impaired tissue repair [10,11]. During chronic inflammation, excessive production of reactive oxygen species (ROS) disrupts tight junction organization, promotes the activation of major pro-inflammatory signaling pathways, and impairs the ability of the intestinal epithelium to efficiently restore its integrity following injury [6,10]. Because impaired epithelial restitution delays mucosal healing, a major therapeutic goal in IBD [5,6], strategies capable of modulating redox homeostasis while supporting epithelial repair are of particular interest [6,12].

In this context, interest in plant-derived bioactive compounds has grown considerably because of their ability to simultaneously modulate several biological processes involved in intestinal homeostasis [13]. Rather than focusing exclusively on individual phenolic compounds, increasing attention is being directed toward standardized plant complexes, whose biological activity may result from additive or synergistic interactions among multiple constituents [14,15] and may encompass the modulation of oxidative stress, inflammatory responses, and epithelial barrier function [13,15].

Among these plant-derived resources, *Citrus* processing by-products, particularly *Citrus* pomace, have attracted increasing attention. *Citrus* pomace, which primarily comprises peel, residual pulp, and seeds, is an abundant source of flavanones and other bioactive phytochemicals, and its valorization is fully consistent with circular-economy principles [16,17]. *Citrus*-derived plant complexes have been reported to exert antioxidant, anti-inflammatory, and cytoprotective effects, supporting their potential development as sustainable food-grade ingredients for gut health applications [18,19]. Nevertheless, evidence concerning food-grade *Citrus* plant complexes obtained through reproducible extraction procedures and characterized by well-defined phytochemical profiles remains limited [15].

Importantly, the potential contribution of *Citrus*-derived plant complexes to intestinal health is not limited to their direct effects on epithelial cells, since the gut microbiota also contributes to the regulation of epithelial permeability, immune function, and metabolism [20,21]. Polyphenols and other plant-derived metabolites engage in a bidirectional interaction with the gut microbiota. They are biotransformed by intestinal microorganisms into biologically active metabolites and, in turn, may selectively influence the growth and metabolic activity of beneficial bacteria [13,22]. From this perspective, assessing the interactions between plant complexes and selected probiotic strains represents an important complement to epithelial cell-based investigations, enabling a more comprehensive understanding of their role as modulators of gut health [13].

In our previous work, standardized food-grade hydroalcoholic extracts obtained from blond orange (OE) and organic lemon (LE) pomace were comprehensively characterized in terms of their phytochemical composition and biological effects in an intestinal epithelial model [23]. Specifically, the preparation and phytochemical characterization of OE and LE, their standardized simulated gastrointestinal digestion, and the characterization of the corresponding digestates were reported in that study [23]. Both OE and LE showed a high content of flavanones and other bioactive phytochemicals and substantially preserved their phytochemical profiles after simulated gastrointestinal digestion. Moreover, the corresponding digestates protected LPS-stimulated Caco-2 cells against oxidative stress, inflammation, and epithelial barrier disruption, highlighting their potential as nutraceutical ingredients aimed at supporting gut health [23]. The previously published biological investigation specifically focused on intracellular oxidative stress, inflammatory gene expression, and epithelial barrier-associated endpoints [23], whereas epithelial restitution, extracellular protein levels, and interactions with selected beneficial bacterial strains were not investigated.

Therefore, the present study was designed to extend our previous findings through experimental endpoints not investigated in the earlier work. The central working hypothesis was that standardized *Citrus* pomace-derived plant complexes would retain measurable biological activity following simulated gastrointestinal digestion and exert complementary effects at the intestinal epithelial and selected microbial levels. After defining a non-cytotoxic concentration range, the ability of OE and LE digestates to promote epithelial wound closure and to concentration-dependently modulate the extracellular levels of oxidative stress-related proteins and pro-inflammatory cytokines was investigated in Caco-2 cells. In this framework, the viability assays established non-cytotoxic exposure conditions, the scratch assay assessed epithelial restitution following mechanical injury, and the LPS-challenged differentiated Caco-2 model was used to evaluate extracellular redox- and inflammation-related responses. In parallel, the effects of both the undigested extracts and their corresponding digestates on selected probiotic strains were preliminarily evaluated by assessing bacterial growth and cell-surface hydrophobicity. These microbiological assays were intended to investigate strain-specific interactions with selected beneficial bacteria before and after simulated gastrointestinal digestion. Together, these complementary experimental approaches were designed to provide an integrated in vitro assessment of distinct epithelial and microbial responses to *Citrus* pomace-derived plant complexes, without implying a common underlying mechanism or direct extrapolation to the complex in vivo intestinal environment.

## 2. Materials and Methods

### 2.1. Cell-Based Models and Biological Assays

#### 2.1.1. Caco-2 Cell Culture and Treatment Conditions

Human colorectal adenocarcinoma Caco-2 cells (ATCC^®^ HTB-37™, American Type Culture Collection, Manassas, VA, USA) were routinely maintained in Dulbecco’s Modified Eagle’s Medium (DMEM; Merck KGaA, Darmstadt, Germany) supplemented with 10% fetal bovine serum (FBS), 1% minimum essential medium non-essential amino acids (MEM-NEAA) and 1% penicillin–streptomycin (Sigma-Aldrich, Merck KGaA, Darmstadt, Germany). Cells were cultured at 37 °C in a humidified atmosphere containing 5% CO_2_. Cell monolayers were subcultured upon reaching 80–90% confluence using trypsin–ethylenediaminetetraacetic acid (trypsin–EDTA). The culture medium was replaced three times per week, and cell cultures were regularly screened for mycoplasma contamination using the Venor^®^ GeM Advance Mycoplasma Detection Kit (Minerva Biolabs, Berlin, Germany) according to the manufacturer’s instructions. Only mycoplasma-free cultures were used throughout the study. To minimize potential passage-related phenotypic changes, all experiments were performed using cells within a predefined passage range (passages 23–32). Experimental treatments were prepared directly in complete culture medium, as described in detail in the corresponding subsections.

#### 2.1.2. MTT Cell Viability Assay

Cell viability was evaluated using the 3-(4,5-dimethylthiazol-2-yl)-2,5-diphenyltetrazolium bromide (MTT) assay, as previously described by Smeriglio et al. [24], with minor modifications. Caco-2 cells were seeded into 96-well plates at a density of 1 × 10^5^ cells per well in quintuplicate and allowed to attach for 24 h at 37 °C in a humidified atmosphere containing 5% CO_2_. The culture medium was then replaced with fresh complete DMEM containing OE or LE digestates, obtained according to the standardized INFOGEST simulated gastrointestinal digestion procedure as previously described [23], at final concentrations ranging from 0.78 to 400 µg/mL. Stock solutions of both digestates were prepared in DMSO and diluted in complete culture medium to achieve a final DMSO concentration of 0.1% (*v*/*v*) in all experimental conditions. Complete DMEM containing 0.1% DMSO served as the vehicle control (CTR−). Following 24 and 48 h of treatment, the culture medium was removed and replaced with MTT solution prepared in DMEM at a final concentration of 0.25 mg/mL, and the cells were incubated for a further 4 h at 37 °C. The MTT solution was then discarded, the cells were gently washed with PBS, and the resulting formazan crystals were dissolved in acidified isopropanol (1 N HCl in isopropanol, 1:24, *v*/*v*). Absorbance was measured at 570 nm using a Varioskan™ LUX multimode microplate reader (Thermo Fisher Scientific, Waltham, MA, USA). Cell-free wells containing the corresponding treatment solutions were included to correct for potential treatment-related background absorbance. Cell viability was expressed as a percentage of the vehicle-treated control.

#### 2.1.3. Scratch Assay

The effects of OE and LE digestates on wound closure were evaluated using an in vitro scratch assay, as previously described by Smeriglio et al. [24], with minor modifications. Caco-2 cells at passage 25 were seeded into 24-well plates at a density of 5 × 10^4^ cells per well in 500 µL of complete DMEM. After the cells had reached a confluent monolayer (72 h after seeding), a linear wound was generated across the center of each monolayer using a sterile 200 µL pipette tip. Detached cells were removed by gently washing the monolayers with PBS, after which 500 µL of fresh complete DMEM containing OE or LE digestates at final concentrations ranging from 0.78 to 400 µg/mL, 0.1% sodium hyaluronate as the positive control (CTR+), or 0.1% DMSO as the vehicle control (CTR−) was added. The full 0.78–400 µg/mL concentration range was initially explored as a screening step. Based on the resulting response profile, 6.25, 12.5, and 25 µg/mL were subsequently selected for quantitative analysis because this interval most clearly captured the concentration-dependent increase in wound closure, spanning responses from those approaching the vehicle control to those within the range observed for the positive control. The concentration range was selected based on the MTT assay, which showed no detectable cytotoxicity across the tested concentrations. Cells were then incubated at 37 °C in a humidified atmosphere containing 5% CO_2_. Wound images were acquired immediately after scratching (0 h) and after 24 and 48 h using an inverted phase-contrast microscope equipped with a digital camera (Leica DMi1, FLEXACAM C1, 12 MP; Leica Camera AG, Wetzlar, Germany) at 10× magnification. Wound closure was quantified using Capture 2.4 software (New York Microscope Company, Hicksville, NY, USA) and expressed as the percentage reduction in wound area relative to the initial wound area measured at 0 h.

#### 2.1.4. Pro-Inflammatory Treatment of Differentiated Caco-2 Monolayers

Unlike the confluent monolayers cultured in conventional 24-well plates for the scratch assay, the inflammatory experiments were performed using fully differentiated Caco-2 monolayers cultured on permeable Transwell inserts, to evaluate treatment responses under polarized epithelial conditions. Caco-2 cells were seeded on 0.4 µm polyethylene terephthalate (PET) membrane inserts (Greiner Bio-One, Kremsmünster, Austria) placed in 24-well plates at a density of 1.056 × 10^5^ cells per well and maintained in complete DMEM until full differentiation was achieved at least 21 days after seeding, with the culture medium replaced three times per week. Before treatment, monolayer integrity was assessed by transepithelial electrical resistance (TEER) using a Millicell ERS-2 volt-ohmmeter (Merck Millipore, Burlington, MA, USA). Only differentiated monolayers exhibiting TEER values ≥ 400 Ω·cm^2^ were included in the study.

Cells were treated via the apical compartment for 24 h with 0.1% DMSO as the vehicle control (CTR−), lipopolysaccharide (LPS; 25 µg/mL) as the pro-inflammatory control (CTR+), OE or LE digestates alone at 200 µg/mL, or LPS in combination with OE or LE digestates at final concentrations of 25, 50, 100, and 200 µg/mL. The LPS concentration of 25 µg/mL was selected based on the dose-ranging experiments performed in the previous study [23], in which 1, 10, and 25 µg/mL LPS were tested in Caco-2 cells. Among these concentrations, 25 µg/mL produced the most pronounced response while inducing only a limited reduction in cell viability (approximately 15%) and was therefore considered suitable for establishing an inflammatory challenge without excessive cytotoxicity. The same concentration was retained in the present study to ensure continuity with the previously characterized experimental model. The concentrations selected for the co-treatment experiments were within the non-cytotoxic range identified by the MTT assay. The upper concentration of 200 µg/mL was selected on the basis of our previous study [23], in which OE and LE digestates at this concentration were shown to counteract LPS-induced inflammatory responses, including the upregulation of IL-1β, IL-6, IL-8, and TNF-α gene expression, while also modulating antioxidant-related genes. Lower concentrations were therefore included in the present study to determine whether these effects exhibited a concentration-dependent pattern. The highest concentration of each digestate was also tested in the absence of LPS to verify that the treatments did not independently induce a pro-inflammatory response. The basolateral compartment received fresh complete DMEM throughout the treatment period. At the end of the 24 h treatment, conditioned media from the apical and basolateral compartments were collected separately and subsequently analyzed independently by ELISA, without pooling the two compartments.

#### 2.1.5. ELISA-Based Quantification of Oxidative Stress and Inflammatory Biomarkers 

Following the treatment conditions described in Section 2.1.4, conditioned media from the apical and basolateral compartments were collected separately after 24 h. Samples were centrifuged at 1000× *g* for 10 min at 4 °C to remove cellular debris, aliquoted, and stored at −80 °C until analysis.

The extracellular levels of the oxidative stress-related proteins SOD2, catalase and Nrf2, as well as those of the pro-inflammatory cytokines IL-1β, IL-6, IL-8 and TNF-α, were quantified using commercially available enzyme-linked immunosorbent assay (ELISA) kits according to the manufacturers’ instructions. SOD2, catalase, and Nrf2 were quantified using the Human SOD2 ELISA Kit (Cat. No. ELH-SOD2), Human Catalase ELISA Kit (Cat. No. ELH-CAT), and the Human Nrf2 ELISA Kit (Cat. No. ELH-NRF2), respectively (RayBiotech, Peachtree Corners, GA, USA). Pro-inflammatory cytokines were quantified using the Human IL-1β ELISA Kit (Cat. No. RA80273-1KT, Sigma-Aldrich, Merck KGaA, Darmstadt, Germany), Human IL-6 ELISA Kit (Cat. No. EZIL6-98K, MilliporeSigma, Burlington, MA, USA), Human IL-8 ELISA Kit (Cat. No. EZHIL8-100K, MilliporeSigma, Burlington, MA, USA) and Human TNF-α ELISA Kit (Cat. No. EZHTNFA-150K, MilliporeSigma, Burlington, MA, USA).

Briefly, standards and samples were added to the corresponding antibody-coated wells and processed according to the protocol supplied with each kit. After the required incubation and washing steps, the appropriate detection and horseradish peroxidase-conjugated reagents were added, as specified by the respective manufacturer. Color development was achieved using tetramethylbenzidine (TMB) substrate, and the enzymatic reaction was terminated by adding the supplied acidic stop solution. Absorbance was measured at 450 nm with a reference wavelength of 620 nm using the Varioskan™ LUX multimode microplate reader described in Section 2.1.2. Analyte concentrations were calculated from the corresponding standard curves using the curve-fitting procedure recommended by each manufacturer and expressed as ng/mL for SOD2 and catalase, and as pg/mL for Nrf2, IL-1β, IL-6, IL-8, and TNF-α.

#### 2.1.6. Assessment of Differentiated Monolayer Viability Using the MTT Assay

The viability of differentiated Caco-2 monolayers was evaluated using the MTT colorimetric assay. Following the experimental design described in Section 2.1.4, monolayers were exposed for 24 h to 0.1% DMSO as the vehicle control (CTR−), 25 µg/mL LPS as the pro-inflammatory control (CTR+), OE and LE digestates alone (200 µg/mL), or LPS in combination with OE or LE digestates at final concentrations of 25, 50, 100 and 200 µg/mL.

After 24 h of incubation, the treatment solutions were removed, and the monolayers were washed with PBS. Subsequently, 900 µL of MTT solution prepared in PBS at a concentration of 1 mg/mL (Merck KGaA, Darmstadt, Germany) was added to the basolateral compartment, whereas no reagent was added to the apical compartment. The plates were incubated for 3 h at 37 °C in a humidified atmosphere containing 5% CO_2_. At the end of the incubation, the MTT solution was removed, and 1.5 mL of isopropanol was added in the basolateral compartment to dissolve the resulting formazan crystals. Complete formazan solubilization was achieved by orbital shaking until a homogeneous solution was obtained. The Transwell inserts were then removed, and absorbance was measured directly in the 24-well plates at 570 nm using the Varioskan™ LUX multimode microplate reader described in Section 2.1.2. Cell viability was expressed as a percentage of the vehicle-treated control.

### 2.2. In Vitro Assessment of the Effects of OE and LE and Their Corresponding Digestates on Selected Probiotic Strains

#### 2.2.1. Growth of Selected Probiotic Strains in the Presence of OE and LE

The effects of OE and LE on the growth of selected probiotic strains were evaluated by spectrophotometric monitoring of bacterial biomass.

Four probiotic lactic acid bacteria (LAB) strains were used in this study: *Lactobacillus gasseri* LG050, *Lactiplantibacillus plantarum* 299V, *Lacticaseibacillus rhamnosus* GG, and *Lactobacillus delbrueckii* subsp. *bulgaricus*, all obtained from commercial formulations. The strains were selected as commercially established LAB representative of lactobacilli commonly used in food and probiotic formulations, with the aim of providing a preliminary assessment of the interactions between *Citrus*-derived plant complexes and selected beneficial bacterial strains.

The strains were reactivated and cultured in de Man, Rogosa and Sharpe (MRS) broth for 16–18 h at 37 °C, except for *L. plantarum* 299V, which was incubated at 30 °C under otherwise identical conditions.

OE and LE were added at a concentration corresponding to 5 µg GAE/mL, equivalent to approximately 243 µg/mL and 289 µg/mL of dry OE and LE, respectively, based on their previously determined total phenolic contents. GAE was used here as an analytical standardization parameter for total phenolic content rather than as a measure of the concentration of individual phenolic compounds. This concentration was used as a standardized in vitro exposure to enable comparison between the two plant complexes and their corresponding digestates and should not be interpreted as a physiologically validated estimate of the concentration achieved in vivo in the intestinal lumen.

Bacterial cultures were inoculated into the supplemented media and incubated under their respective optimal growth conditions. Parallel cultures grown in unsupplemented MRS broth were used as controls.

Bacterial growth was evaluated by measuring the optical density at 600 nm (OD600) using a Cary 50 Bio UV–Visible spectrophotometer (Varian, Palo Alto, CA, USA), after 16–18 h of incubation. Results were expressed as bacterial growth (%) relative to the corresponding untreated control, with cultures grown in unsupplemented MRS broth set at 100%.

#### 2.2.2. Growth of Selected Probiotic Strains in the Presence of OE and LE Digestates

To evaluate whether simulated gastrointestinal digestion affected the growth-promoting activity observed for the extracts, OE and LE digestates were added to MRS broth at concentrations corresponding to a total polyphenol content of 5 µg GAE/mL. The selected probiotic strains were cultured under the same experimental conditions described in Section 2.2.1., with cultures grown in unsupplemented MRS broth serving as controls.

Bacterial growth was determined by measuring the optical density at 600 nm (OD600) after 16–18 h of incubation using a Cary 50 Bio UV–Visible spectrophotometer (Varian, Palo Alto, CA, USA). Results were expressed as bacterial growth (%) relative to the corresponding untreated control, with cultures grown in unsupplemented MRS broth set at 100%.

#### 2.2.3. Assessment of Bacterial Cell-Surface Hydrophobicity by the Microbial Adhesion to Solvents (MAS) Assay

The cell-surface hydrophobicity of the selected probiotic strains was evaluated after incubation with OE, LE, and their corresponding digestates using the microbial adhesion to solvents (MAS) assay, with xylene as the hydrophobic solvent. The assay was used to assess treatment-induced changes in bacterial cell-surface physicochemical properties rather than as a direct measure of bacterial adhesion to intestinal epithelial cells.

Following bacterial growth under the experimental conditions described above, cells were harvested by centrifugation, washed with sterile 0.9% NaCl solution, and resuspended in the same solution to obtain a bacterial suspension with a standardized optical density at 600 nm (OD600). The initial absorbance of the bacterial suspension (A_0_) was measured at 600 nm.

An equal volume of xylene was then added to each bacterial suspension, and the two-phase system was vigorously vortexed for 3 min. After phase separation for 60 min at the corresponding growth temperature, the aqueous phase was carefully collected, and its absorbance (*A*_1_) was measured at 600 nm. Cell-surface hydrophobicity was calculated according to the following equation:

$$Hydrophobicity\;(\%)\;=\;[(A_{0}\;-\;A_{1})/A_{0}]\;\times \;100$$

where *A*_0_ represents the absorbance of the bacterial suspension before contact with xylene and *A*_1_ the absorbance of the aqueous phase after phase separation.

### 2.3. Statistical Analysis

Data were expressed as the mean ± standard deviation (SD) from three independent experiments. For all analyses, the independent experiment was considered the experimental unit (*n* = 3). Replicate wells or repeated analytical measurements performed within the same independent experiment were considered technical replicates and were averaged to obtain a single value for each experimental condition before statistical testing. Thus, all statistical analyses were performed using the three independent experimental values and not the individual technical replicates. Statistical significance was evaluated using one-way analysis of variance (ANOVA), followed by Student–Newman–Keuls post hoc test for multiple comparisons. Normality of data distribution was assessed using the Shapiro–Wilk test. Homogeneity of variance was assessed using Levene’s test, which confirmed that the homogeneity assumption was satisfied. Differences were considered statistically significant at *p* < 0.05. All statistical analyses were performed using SigmaPlot version 12.0 (Systat Software Inc., San Jose, CA, USA).

## 3. Results

### 3.1. Biological Effects in Cell-Based Models

#### 3.1.1. Effects of OE and LE Digestates on Caco-2 Cell Viability

**Figure 1:**
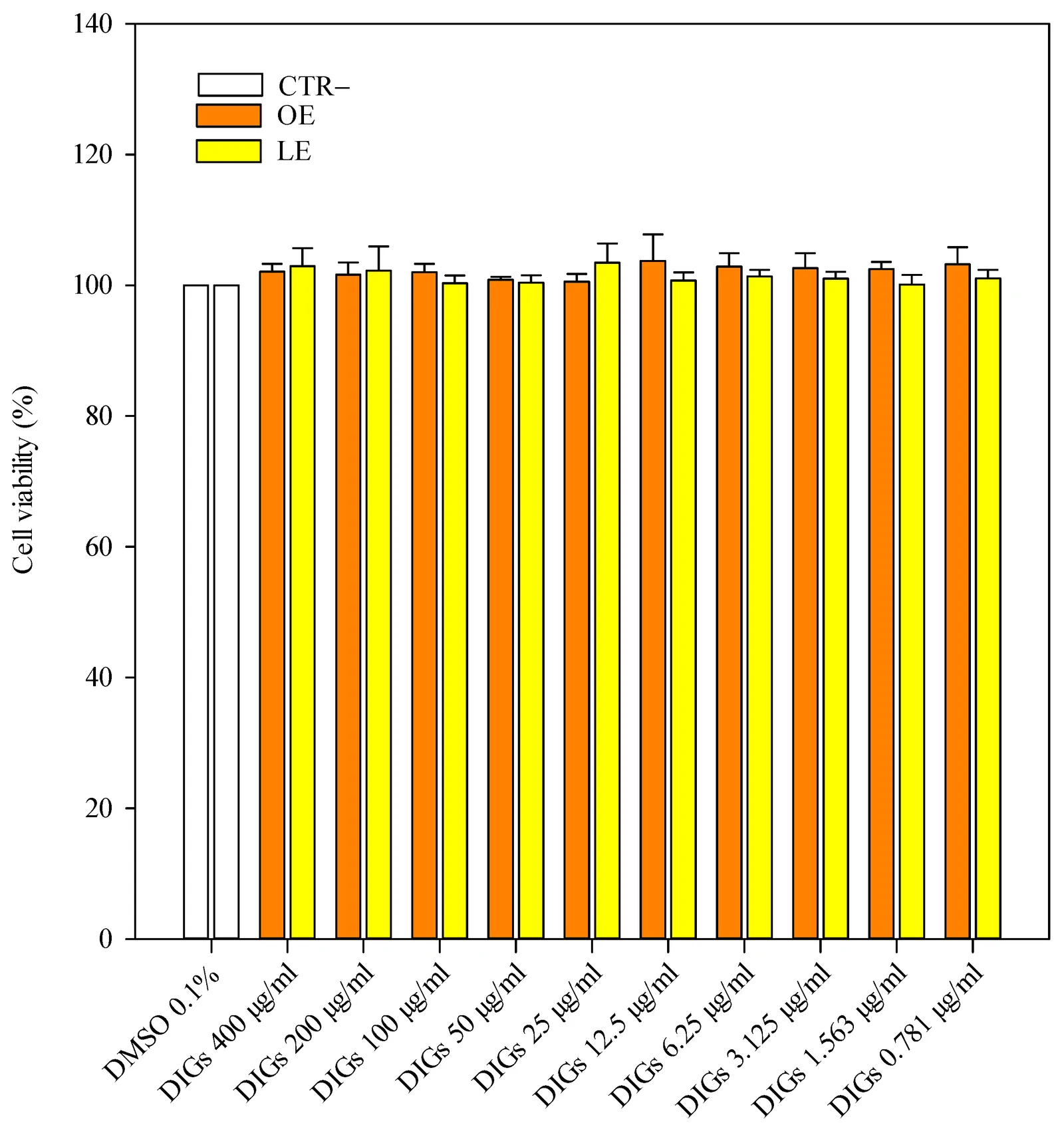
Effects of OE and LE digestates (DIGs) on Caco-2 cell viability after 48 h of treatment. Cells were treated with OE or LE DIGs at concentrations ranging from 0.78 to 400 µg/mL. Cell viability was assessed using the MTT assay and expressed as a percentage relative to the vehicle control (CTR−; 0.1% DMSO). Data are expressed as the mean ± SD of three independent experiments, each performed in quintuplicate (*n* = 3 independent experiments). Statistical analysis was performed using one-way ANOVA followed by the Student–Newman–Keuls post hoc test. No statistically significant differences were observed relative to CTR−.

To identify a non-cytotoxic concentration range for subsequent functional assays, Caco-2 cell viability was assessed following treatment with OE or LE digestates at concentrations ranging from 0.78 to 400 µg/mL. As no statistically significant changes in cell viability were observed at either 24 or 48 h, only data obtained after 48 h, corresponding to the longest exposure time, are shown (Figure 1).

Across the entire concentration range tested, neither OE nor LE digestates significantly affected Caco-2 cell viability relative to the vehicle control (CTR−). Viability remained close to 100% even at the highest concentration tested (400 µg/mL), confirming the absence of detectable cytotoxicity under these experimental conditions. Based on these findings, concentrations of 6.25, 12.5 and 25 µg/mL were selected for the subsequent wound-healing assay.

#### 3.1.2. Assessment of Epithelial Wound Closure Using a Scratch Assay

**Figure 2:**
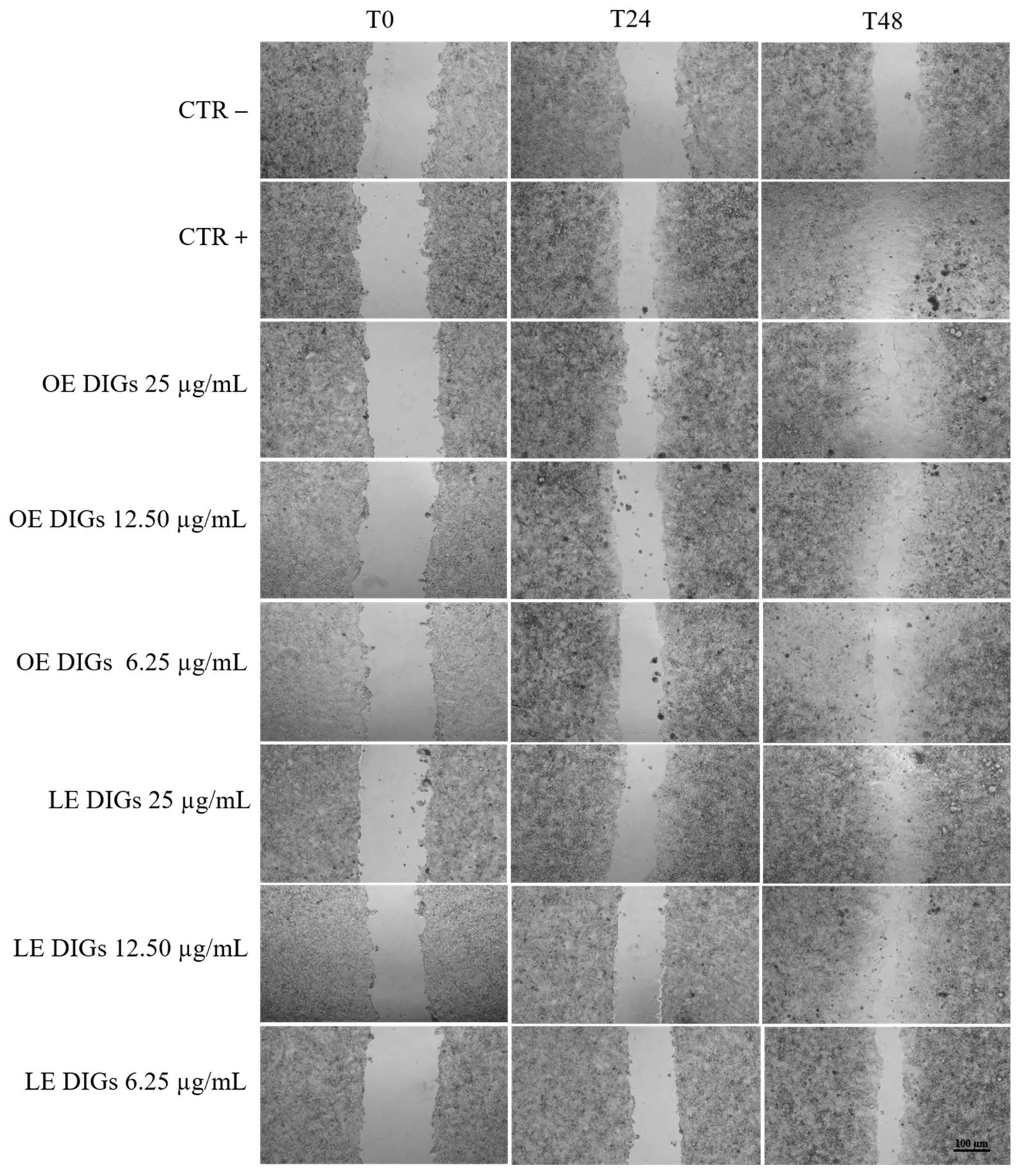
Representative micrographs from the wound-healing assay showing wound closure in confluent Caco-2 monolayers treated with OE or LE digestates (DIGs) at concentrations of 6.25, 12.5, and 25 µg/mL. Cells were treated with 0.1% DMSO as the vehicle control (CTR−), 0.1% sodium hyaluronate as the positive control (CTR+), or OE or LE DIGs. Images were acquired immediately after scratching (T0) and after 24 h (T24) and 48 h (T48) of incubation. Scale bar: 100 µm.

**Figure 3:**
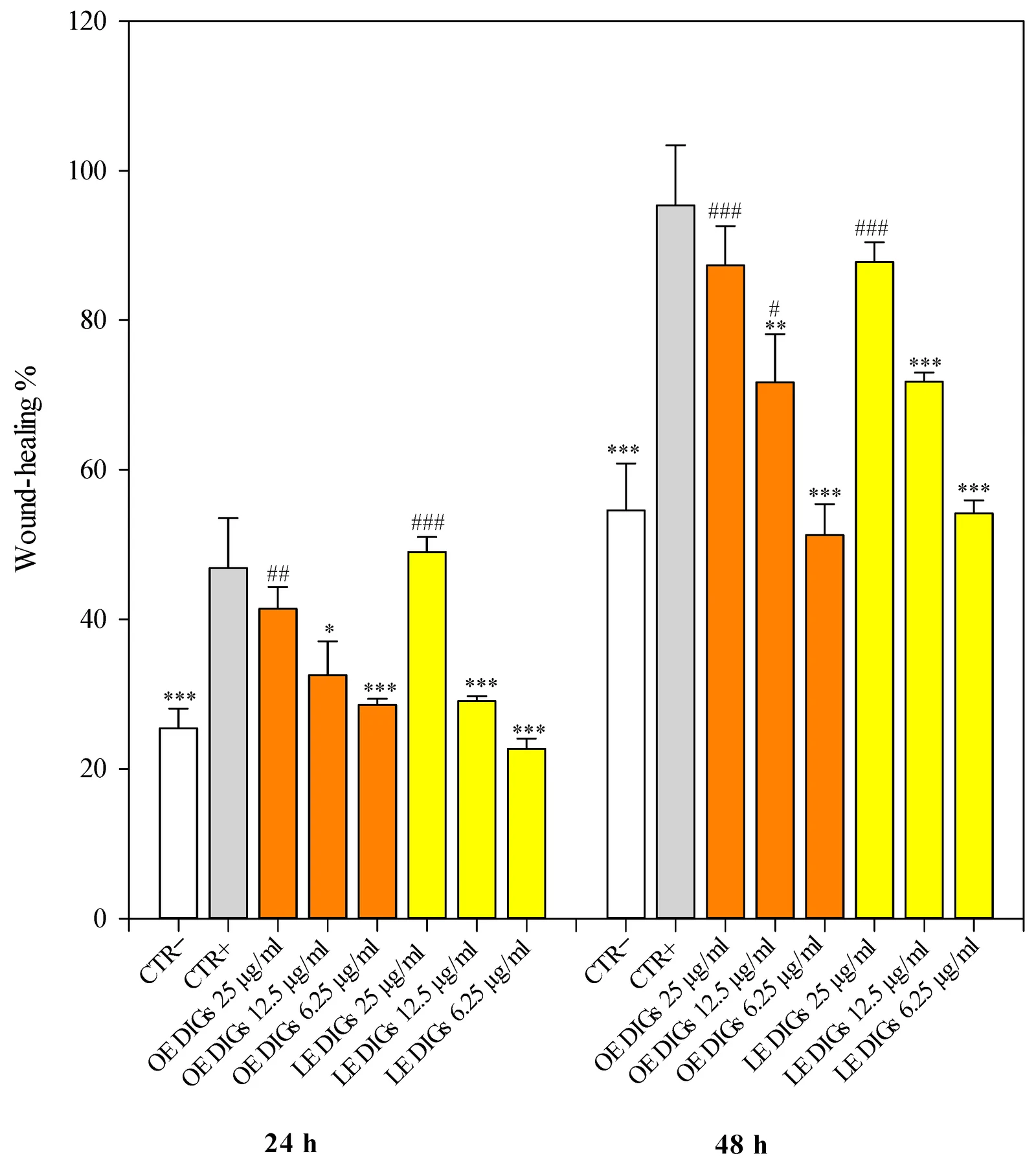
Effect of OE and LE digestates (DIGs) on wound closure in confluent Caco-2 monolayers. Wound closure was assessed at 24 h and 48 h after treatment with OE or LE DIGs at 6.25, 12.5, and 25 μg/mL, 0.1% sodium hyaluronate as the positive control (CTR+), or 0.1% DMSO as the vehicle control (CTR−). Data are expressed as the mean ± SD of three independent experiments, each conducted in triplicate (*n* = 3 independent experiments). Statistical analyses were performed separately for the 24 h and 48 h time points using one-way ANOVA followed by the Student–Newman–Keuls post hoc test for multiple comparisons: * *p* ≤ 0.05, ** *p* ≤ 0.01 and *** *p* ≤ 0.001 vs. CTR+; ^#^ *p* ≤ 0.05, ^##^ *p* ≤ 0.01, and ^###^ *p* ≤ 0.001 vs. CTR−.

To evaluate the effects of OE and LE digestates on epithelial restitution, a wound-healing assay was performed using confluent Caco-2 monolayers. Given the absence of detectable cytotoxicity across the concentration range investigated, the effects of both digestates on wound closure were initially explored over a broader concentration range, up to 400 µg/mL. The 6.25–25 µg/mL range was selected for presentation because it most clearly captured the concentration-dependent response, spanning from effects approaching those of the vehicle control to responses within the range observed for the positive control. Accordingly, the quantitative analysis focused on 6.25, 12.5, and 25 µg/mL. Representative micrographs acquired immediately after scratching (T0) and after 24 h (T24) and 48 h (T48) of treatment are shown in Figure 2, whereas the quantitative analysis of wound closure is reported in Figure 3.

After 24 h of treatment, both digestates promoted wound closure in a concentration-dependent manner. The vehicle control (CTR−) showed a wound closure of 25.4 ± 2.7%, whereas the positive control (CTR+) reached 46.8 ± 6.7%. At 25 µg/mL, wound closure increased to 41.4 ± 2.9% and 49.0 ± 2.0% following treatment with OE and LE digestates, respectively. LE at 25 µg/mL produced a wound-closure response comparable to that of the positive control, whereas lower concentrations were associated with progressively lower values.

After 48 h, wound closure further increased under all experimental conditions. The vehicle control and positive control reached 54.6 ± 6.2% and 95.3 ± 8.1%, respectively. At 25 µg/mL, OE and LE digestates achieved wound closure values of 87.3 ± 5.3% and 87.8 ± 2.6%, respectively, whereas treatment with 12.5 µg/mL resulted in values of 71.7 ± 6.5% and 71.8 ± 1.2%, respectively.

Overall, a progressive increase in wound closure was observed with increasing digestate concentration and incubation time. Both OE and LE digestates enhanced epithelial wound closure, with the most pronounced responses observed at 25 µg/mL.

#### 3.1.3. Effects on Oxidative Stress and Inflammatory Biomarkers 

**Figure 4:**
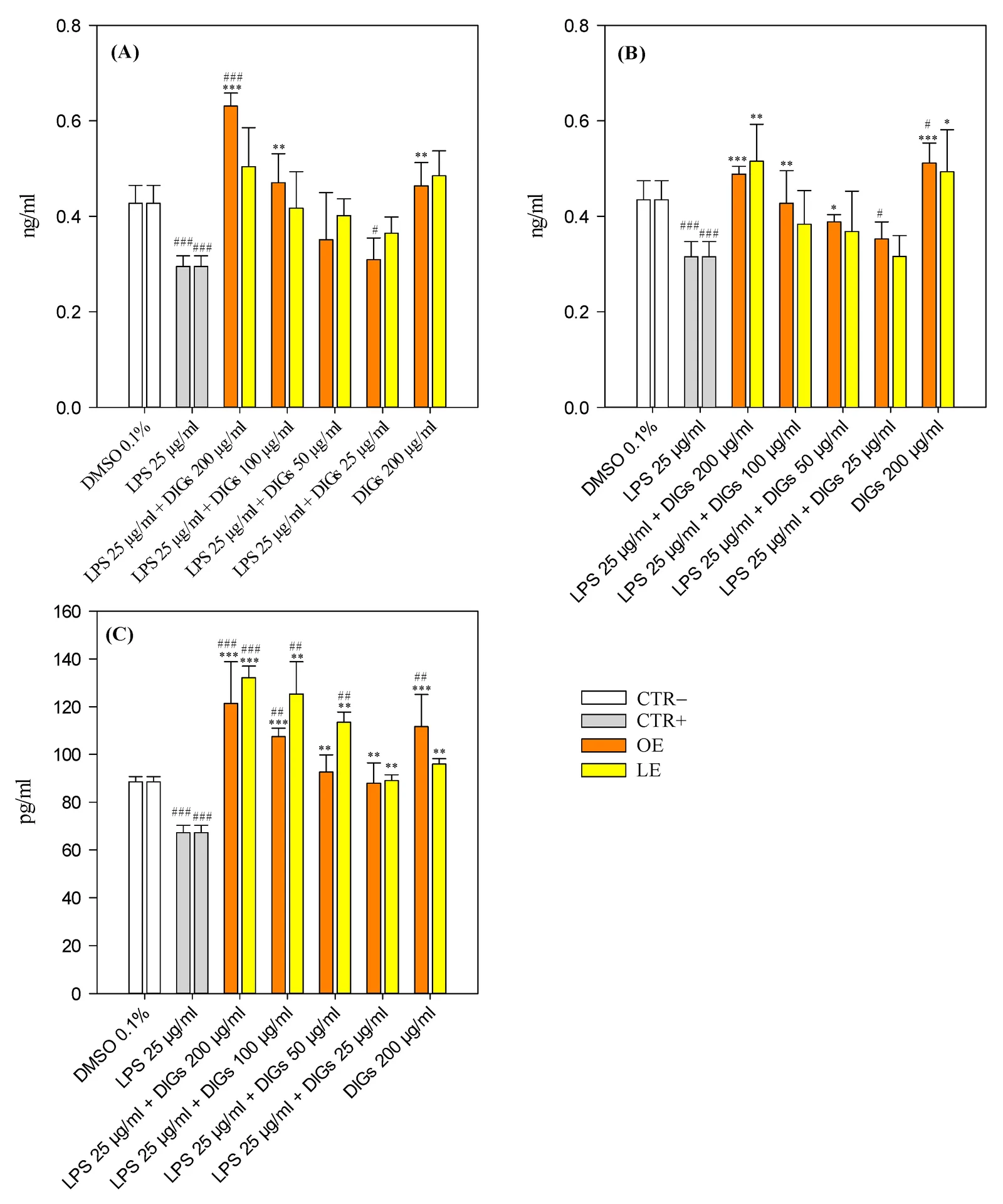
Extracellular levels of (**A**) SOD2, (**B**) catalase, and (**C**) Nrf2 quantified by ELISA. Differentiated Caco-2 monolayers were treated with DMSO (0.1%; vehicle control, CTR−), LPS (25 μg/mL; pro-inflammatory control, CTR+), OE or LE digestates (DIGs) alone (200 μg/mL), or co-treated with LPS and OE or LE DIGs at 25, 50, 100, or 200 μg/mL. Orange bars represent OE DIGs, whereas yellow bars represent LE DIGs. Data are expressed as the mean ± SD of three independent experiments, each conducted in triplicate (*n* = 3 independent experiments). Statistical analysis was performed using one-way ANOVA followed by the Student–Newman–Keuls post hoc test for multiple pairwise comparisons: * *p* ≤ 0.05, ** *p* ≤ 0.01 and *** *p* ≤ 0.001 vs. LPS (25 µg/mL); ^#^ *p* ≤ 0.05, ^##^ *p* ≤ 0.01, and ^###^ *p* ≤ 0.001 vs. DMSO (0.1%).

**Figure 5:**
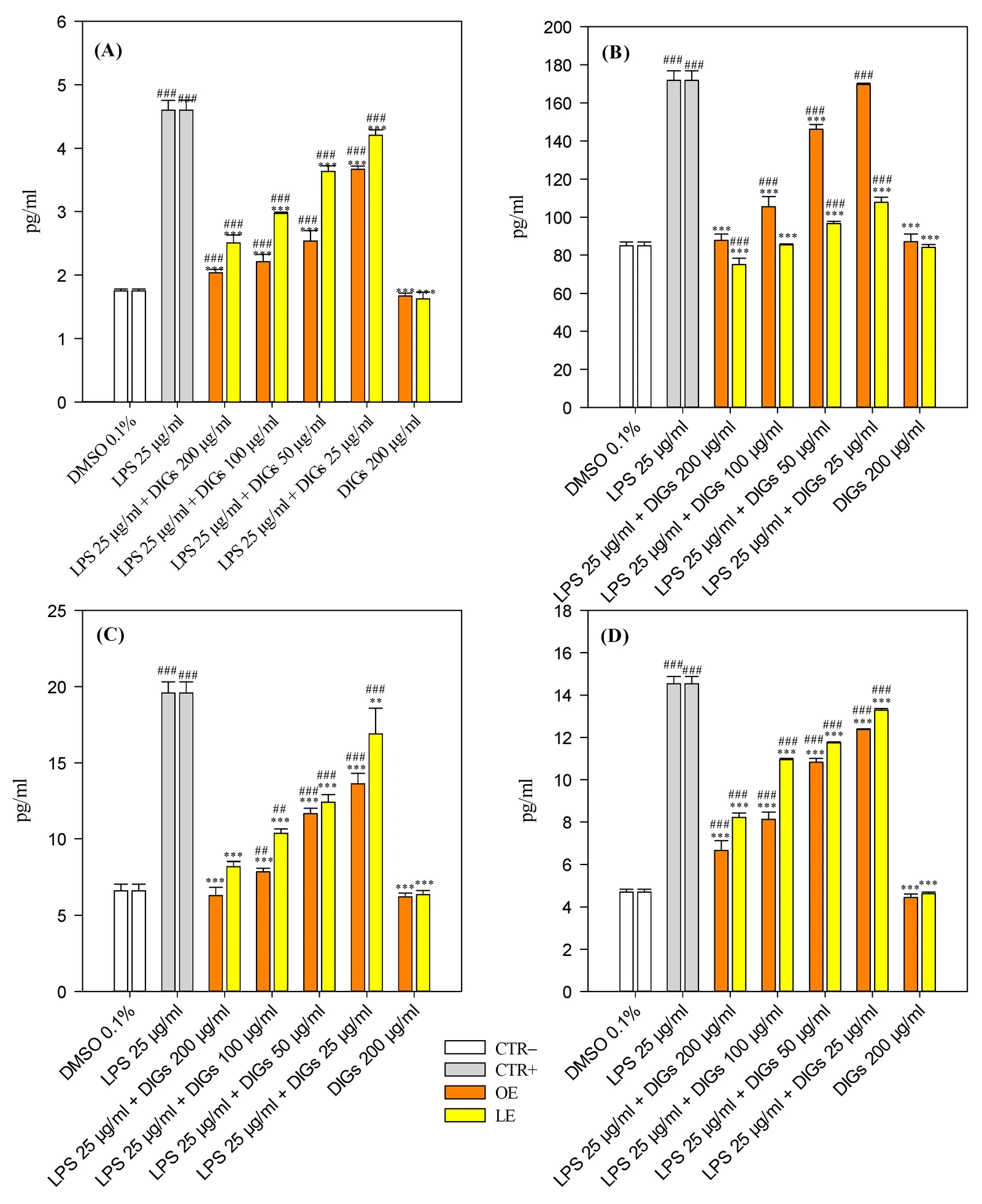
Extracellular levels of (**A**) IL-6, (**B**) IL-8, (**C**) TNF-α, and (**D**) IL-1β quantified by ELISA. Differentiated Caco-2 monolayers were treated with DMSO (0.1%; vehicle control, CTR−), LPS (25 μg/mL; pro-inflammatory control, CTR+), OE or LE digestates (DIGs) alone (200 µg/mL), or co-treated with LPS and OE or LE DIGs at 25, 50, 100 or 200 μg/mL. Orange bars represent OE DIGs, whereas yellow bars represent LE DIGs. Data are expressed as the mean ± SD of three independent experiments, each conducted in triplicate (*n* = 3 independent experiments). Statistical analysis was performed using one-way ANOVA followed by the Student–Newman–Keuls post hoc test for multiple pairwise comparisons: ** *p* ≤ 0.01 and *** *p* ≤ 0.001 vs. LPS (25 µg/mL); ^##^ *p* ≤ 0.01 and ^###^ *p* ≤ 0.001 vs. DMSO (0.1%).

To evaluate the effects of OE and LE digestates on the extracellular levels of oxidative stress- and inflammation-related proteins, SOD2, catalase, and Nrf2, together with the pro-inflammatory cytokines IL-6, IL-8, TNF-α, and IL-1β, were quantified by ELISA in conditioned media collected from differentiated Caco-2 monolayers after 24 h of treatment (Figure 4 and Figure 5).

As shown in Figure 4A–C, LPS significantly reduced the extracellular levels of SOD2, catalase, and Nrf2 compared with the vehicle control (CTR−; 0.1% DMSO). Co-treatment with OE or LE digestates significantly counteracted these LPS-induced reductions in a concentration-dependent manner, with progressively greater attenuation of these reductions as the digestate concentration increased. Treatment with OE or LE digestates alone (200 µg/mL) did not significantly alter the basal extracellular levels of SOD2, catalase, or Nrf2 compared with the vehicle control.

As shown in Figure 5A–D, LPS significantly increased the extracellular levels of IL-6, IL-8, TNF-α, and IL-1β compared with the vehicle control. Co-treatment with OE or LE digestates significantly attenuated the LPS-induced increase in each of the measured cytokines in a concentration-dependent manner, with progressively greater reductions observed at increasing digestate concentrations. Likewise, treatment with OE or LE digestates alone (200 µg/mL) did not significantly affect the basal extracellular levels of these cytokines compared with the vehicle control.

Taken together, these findings show that OE and LE digestates concentration-dependently mitigated the LPS-induced alterations in the extracellular levels of oxidative stress-related proteins and pro-inflammatory cytokines in differentiated Caco-2 monolayers.

#### 3.1.4. Effects of OE and LE Digestates on the Viability of Differentiated Caco-2 Monolayers

**Figure 6:**
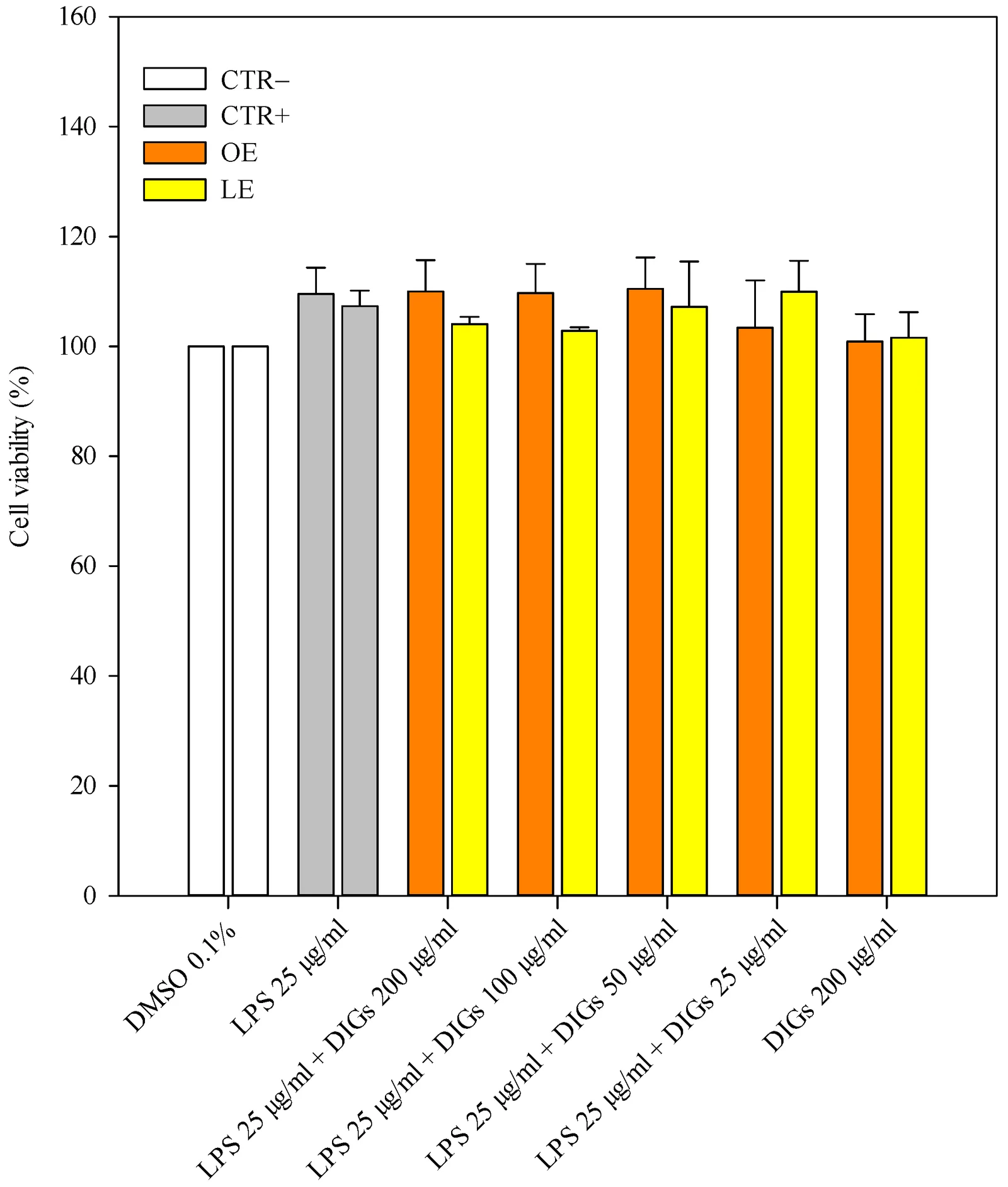
Effects of OE and LE digestates (DIGs) on the viability of differentiated Caco-2 monolayers. Monolayers were treated for 24 h with DMSO (0.1%; vehicle control, CTR−), LPS (25 μg/mL; pro-inflammatory control, CTR+), OE or LE DIGs alone (200 µg/mL), or co-treated with LPS and OE or LE DIGs at 25, 50, 100 or 200 µg/mL. Orange bars represent OE DIGs, whereas yellow bars represent LE DIGs. Cell viability was assessed using the MTT assay and expressed as a percentage relative to CTR−. Data are expressed as the mean ± SD of three independent experiments, each conducted in triplicate (*n* = 3 independent experiments). Statistical analysis was performed using one-way ANOVA followed by the Student–Newman–Keuls post hoc test for multiple pairwise comparisons. No statistically significant differences were observed among the experimental groups.

To determine whether the effects observed on oxidative stress-related proteins and inflammatory mediators were associated with changes in cell viability, differentiated Caco-2 monolayers were evaluated by the MTT assay after 24 h of treatment under the same experimental conditions used for the ELISA analyses (Figure 6).

Neither LPS nor OE or LE digestates, either alone or in combination, significantly affected the viability of differentiated Caco-2 monolayers relative to the vehicle control (CTR−). Cell viability remained comparable across all experimental conditions, indicating the absence of detectable cytotoxicity under the treatment conditions employed. These findings indicate that the observed modulation of extracellular oxidative stress-related proteins and inflammatory mediators was not attributable to treatment-induced changes in cell viability.

### 3.2. Effects of OE and LE and Their Corresponding Digestates on the Growth and Cell-Surface Hydrophobicity of Selected Probiotic Strains

To investigate the effects of OE and LE before and after simulated gastrointestinal digestion on selected probiotic lactic acid bacteria (LAB), bacterial growth and cell-surface hydrophobicity were evaluated in *Lactobacillus delbrueckii* subsp. *bulgaricus*, *Lactobacillus gasseri* LG050, *Lactiplantibacillus plantarum* 299V, and *Lacticaseibacillus rhamnosus* GG.

**Figure 7:**
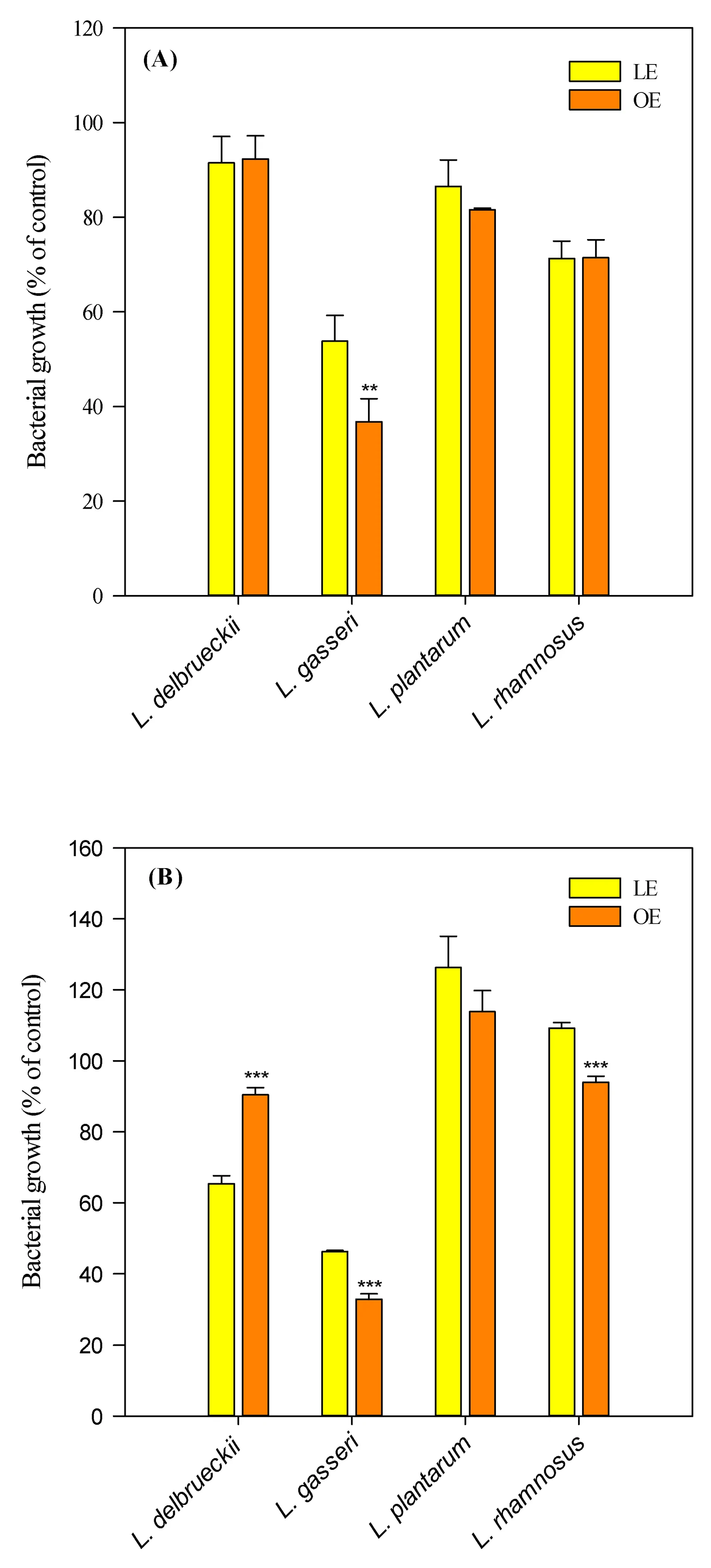
Effects of OE and LE on the growth of selected probiotic strains before (**A**) and after simulated gastrointestinal digestion (**B**). Bacterial growth was assessed by measuring the optical density at 600 nm (OD600) after 16–18 h of incubation in the presence of OE or LE, or their corresponding digestates. Growth was expressed as a percentage relative to the untreated control (CTR), set at 100%. Data are expressed as the mean ± SD of three independent experiments, each conducted in triplicate (*n* = 3 independent experiments). Statistical analysis was performed using one-way ANOVA followed by the Student–Newman–Keuls post hoc test for multiple pairwise comparisons: ** *p* ≤ 0.01 and *** *p* ≤ 0.001 vs. the corresponding LE treatment.

The effects of OE and LE on bacterial growth before and after simulated gastrointestinal digestion are shown in Figure 7.

Before simulated gastrointestinal digestion (Figure 7A), both extracts modulated bacterial growth in a strain-dependent manner. No uniform stimulation of bacterial growth was observed relative to the untreated control. *L. gasseri* exhibited the greatest reduction in growth, particularly following treatment with OE, whereas *L. delbrueckii* subsp. *bulgaricus*, and *L. plantarum* showed more limited changes. Significant differences between OE and LE were observed in selected strains, further supporting a strain-dependent response to the two extracts.

Following simulated gastrointestinal digestion (Figure 7B), the bacterial growth profile changed markedly while retaining a clear strain dependence. In particular, *L. delbrueckii* subsp. *bulgaricus* and *L. plantarum* showed growth values exceeding those observed under the corresponding pre-digestion conditions, whereas *L. gasseri* remained the strain exhibiting the lowest growth values. Significant differences between OE and LE persisted in selected strains, indicating that gastrointestinal digestion modified the strain-specific response to the two plant complexes.

**Figure 8:**
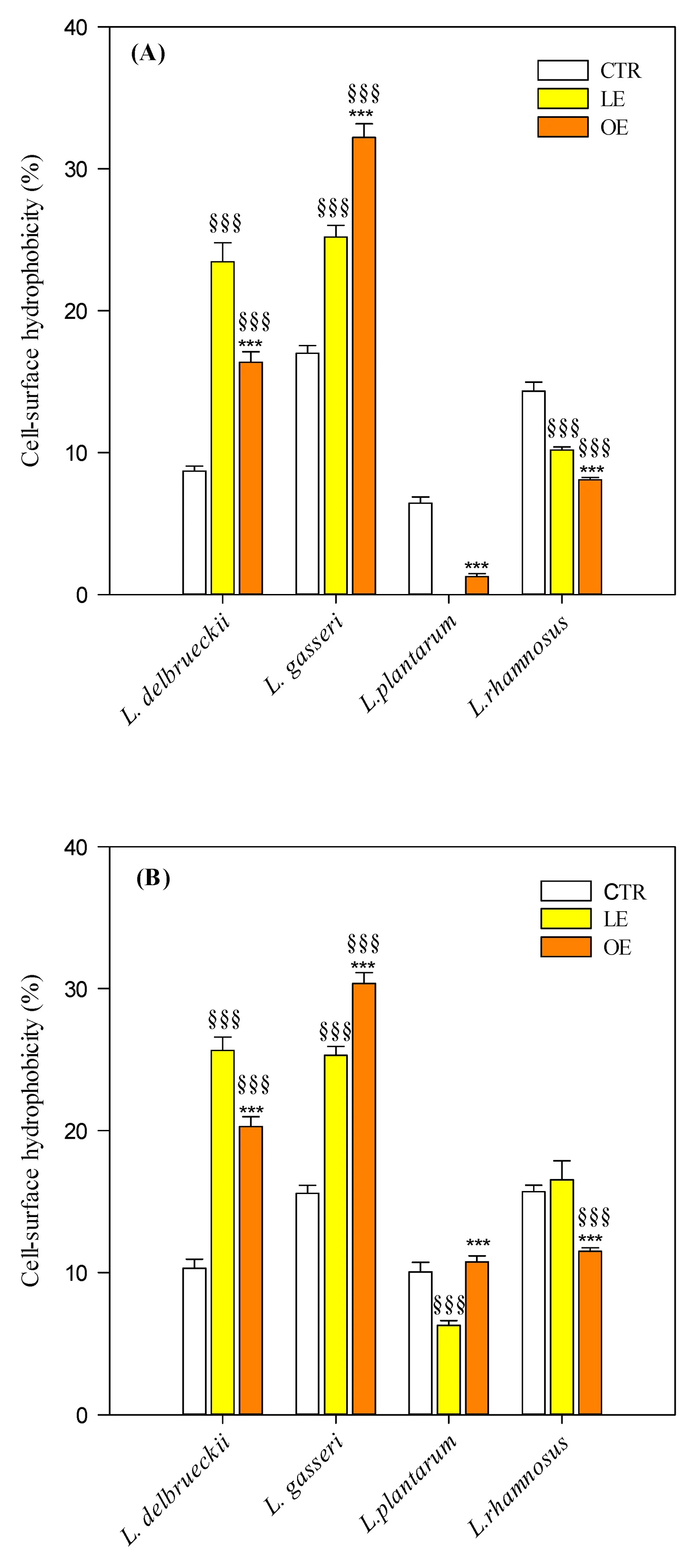
Effects of OE and LE on the cell-surface hydrophobicity of selected probiotic strains before (**A**) and after simulated gastrointestinal digestion (**B**), as assessed using the microbial adhesion to solvents (MAS) assay with xylene. Cell-surface hydrophobicity is expressed as an absolute percentage and was calculated from the decrease in the optical density of the aqueous phase after partitioning with xylene. Data are expressed as the mean ± SD of three independent experiments. Statistical analysis was performed using one-way ANOVA followed by the Student–Newman–Keuls post hoc test for multiple pairwise comparisons: *** *p* ≤ 0.001 vs. the corresponding LE treatment; ^§§§^ *p* ≤ 0.001 vs. the untreated control (CTR).

Cell-surface hydrophobicity was subsequently evaluated using the microbial adhesion to solvents (MAS) assay, and the results are shown in Figure 8.

Before simulated gastrointestinal digestion (Figure 8A), OE and LE significantly modulated cell-surface hydrophobicity in a strain-dependent manner. Increased hydrophobicity relative to the untreated control was observed for *L. delbrueckii* subsp. *bulgaricus* and *L. gasseri*, whereas the response of *L. plantarum* and *L. rhamnosus* was characterized by either limited or reduced hydrophobicity depending on the treatment. Significant differences between the two extracts were also detected in specific strains, indicating distinct effects of OE and LE on bacterial surface properties.

Following simulated gastrointestinal digestion (Figure 8B), cell-surface hydrophobicity remained strongly strain-dependent, although the response profile differed from that observed with the undigested extracts. Both digestates increased hydrophobicity in *L. delbrueckii* subsp. *bulgaricus* and *L. gasseri* relative to the untreated control, whereas *L. plantarum* and *L. rhamnosus* displayed more variable responses, including reductions under selected treatment conditions. Significant differences between OE and LE persisted in selected strains, suggesting that gastrointestinal digestion altered the effects of the plant complexes on bacterial cell-surface properties. Overall, OE and LE, as well as their corresponding digestates, produced highly strain-specific effects on both bacterial growth and cell-surface hydrophobicity, with the latter showing particularly marked treatment-dependent variations. 

## 4. Discussion

The present study extends our previous investigation on standardized food-grade *Citrus* pomace-derived plant complexes by providing additional evidence of their biological activity following simulated gastrointestinal digestion. Whereas the previous study characterized their effects on intracellular oxidative stress, redox- and inflammation-related signaling, and epithelial barrier-associated endpoints in LPS-stimulated Caco-2 cells [23], the present work extends this characterization to additional functional endpoints, namely Caco-2 wound closure, extracellular redox- and inflammation-related proteins, and interactions with selected probiotic strains. Furthermore, these findings provide additional support for the valorization of standardized plant complexes recovered from *Citrus* processing by-products as food-grade ingredients with potential nutraceutical applications, while contributing to the sustainable valorization of agro-industrial residues [25,26].

An additional strength of the present study lies in the use of the bioaccessible fraction obtained after standardized simulated gastrointestinal digestion. Since dietary polyphenols undergo physicochemical modifications during gastrointestinal transit, evaluating digestates rather than native extracts provides a more physiologically relevant assessment of the compounds potentially available to interact with the intestinal epithelium and, subsequently, with intestinal microorganisms. The internationally harmonized INFOGEST protocol also improves the reproducibility and comparability of the findings with those of other studies [16,27]. Nevertheless, simulated digestion remains an in vitro approximation and cannot reproduce the dynamic physiological, metabolic, and microbial processes occurring in vivo.

One of the main findings of the present study was the ability of both OE and LE digestates to enhance wound closure in confluent Caco-2 monolayers under non-cytotoxic conditions. Rapid closure of epithelial discontinuities is an important component of the response to mucosal injury [28], whereas persistent impairment of epithelial repair contributes to barrier dysfunction in IBD and is clinically related to the broader concept of mucosal healing [29,30]. Within the limits of the present in vitro model, the observed effect therefore complements the epithelial barrier-associated activity previously reported for OE and LE digestates [23].

The concentration- and time-dependent response observed in the scratch assay is consistent with previous reports describing protective effects of *Citrus*-derived flavanones on intestinal epithelial integrity and barrier function [16,18,19]. However, the present findings should be interpreted specifically as enhanced wound closure in a Caco-2 monolayer rather than as a demonstration of complete intestinal epithelial repair or mucosal healing.

Nevertheless, the activity observed here is unlikely to be attributable to a single phytochemical. OE and LE are standardized plant complexes containing multiple flavanones together with other phenolic constituents that remain bioaccessible after simulated gastrointestinal digestion. Increasing evidence indicates that complex botanical preparations can exert multitarget biological effects through additive, synergistic, or otherwise complementary interactions among their constituents, thereby more closely reflecting the physiological complexity of dietary phytochemicals than isolated compounds [15,18,31]. Accordingly, the wound-closure activity observed in the present study is more appropriately interpreted as an effect of the overall bioaccessible plant complex rather than being assigned to any individual constituent.

Some methodological considerations should also be acknowledged. Although the scratch assay is widely used to investigate wound-closure, wound closure reflects the combined contribution of collective cell migration, cell proliferation, and cytoskeletal remodeling. Since no proliferation inhibitor was included in the experimental design, the relative contribution of these processes cannot be distinguished. Furthermore, this model does not reproduce the complexity of the intestinal mucosa, where epithelial cells continuously interact with immune cells, stromal components, and the resident microbiota. Therefore, although the present findings provide clear evidence of enhanced wound closure following treatment with OE and LE digestates in vitro, additional mechanistic studies using more complex experimental models will be required to clarify the cellular pathways involved.

The extracellular protein measurements provided a complementary level of evidence to the intracellular and transcriptional responses characterized in the previous study. Under LPS challenge, OE and LE digestates concentration-dependently attenuated alterations in extracellular oxidative stress-related proteins and pro-inflammatory cytokines without affecting cell viability, excluding cytotoxicity as an explanation for these changes.

Oxidative stress and inflammation are closely interconnected in the pathogenesis of IBD. Their reciprocal amplification provides a biological rationale for considering redox and inflammatory responses together rather than as independent processes [32].

Nrf2-Keap1 signaling plays a central role in cellular antioxidant defense and intestinal homeostasis [33,34] and is functionally interconnected with pro-inflammatory pathways, including NF-κB [35]. Importantly, modulation of these intracellular redox- and inflammation-related responses had already been demonstrated in the previous study, which showed effects of OE and LE digestates on intracellular oxidative stress, antioxidant- and inflammation-related gene expression, including CAT, NRF2 and SOD2, and pNF-κB p65 nuclear translocation, together with epithelial barrier-associated endpoints [23]. The present extracellular measurements were therefore not intended to independently re-establish those signaling mechanisms, but rather to determine whether the previously documented intracellular and transcriptional responses were accompanied by corresponding concentration-dependent changes at the extracellular protein level. Because SOD2, catalase, and Nrf2 were quantified in conditioned media, these measurements should nevertheless be interpreted as complementary protein-level evidence and not as direct intracellular readouts of pathway activity. Taken together, the two studies provide complementary evidence across intracellular, transcriptional, extracellular protein, and functional endpoints, while avoiding attribution of the present extracellular measurements to a mechanism beyond that directly investigated in the current experimental design. Maintenance of intestinal homeostasis, however, depends not only on epithelial responses but also on interactions between dietary plant complexes and the resident microbiota. Accordingly, the present study also investigated the effects of OE and LE, before and after simulated gastrointestinal digestion, on selected probiotic strains. Dietary polyphenols and gut microorganisms have a bidirectional relationship: microorganisms metabolize phytochemicals into bioactive metabolites, while phytochemicals selectively influence microbial composition and function [22]. Within a strain-specific in vitro screening, the response of selected probiotic bacteria therefore provides complementary information on potential interactions between these microorganisms and the *Citrus*-derived plant complexes [16,22,36]. Nevertheless, the present screening was restricted to selected probiotic LAB, and including other beneficial bacterial groups, particularly *Bifidobacterium* spp., would broaden the microbiological characterization of these plant complexes.

Consistent with previous evidence, OE and LE and their corresponding digestates elicited markedly strain-dependent growth responses. Such variability likely reflects differences in bacterial metabolic capabilities, enzymatic repertoires, and substrate utilization, which determine the ability of individual microorganisms to metabolize flavonoids and other phenolic compounds [16,36]. These findings therefore argue against a generalized growth-promoting effect and instead support selective interactions between the *Citrus* plant complexes and individual bacterial strains. It should also be acknowledged that bacterial growth was assessed by endpoint OD600 measurements, which provide a comparative estimate of culture turbidity but do not distinguish viable from non-viable cells or provide information on growth kinetics. Therefore, future studies combining growth-curve analysis with viable cell enumeration will be required to further characterize and confirm the strain-specific responses observed here.

Simulated gastrointestinal digestion further influenced these interactions. Although our previous phytochemical characterization demonstrated that the major flavanones remained detectable and largely preserved after digestion, the gastrointestinal process can modify both the chemical environment and the relative bioaccessibility of individual constituents within the plant complex. Accordingly, the different bacterial responses observed before and after digestion may reflect changes in the bioaccessible fraction and physicochemical state of compounds available for bacterial interaction and metabolism, rather than major alterations in the overall phytochemical profile [16,23].

Changes in cell-surface hydrophobicity were not always associated with corresponding changes in bacterial growth, suggesting that these plant complexes may influence bacterial surface characteristics independently, at least in part, of their effects on growth. However, bacterial surface hydrophobicity is only one of several physicochemical properties that may be involved in bacterial adhesion, and the MAS assay should therefore be considered an indirect indicator of bacterial surface behavior rather than a direct measure of adhesion to the intestinal epithelium. Consequently, these findings should not be interpreted as evidence of improved intestinal colonization but rather as an indication that *Citrus* plant complexes can modulate bacterial surface properties in a strain-dependent manner [37,38]. Direct adhesion assays using intestinal epithelial cell models will be required to determine whether these treatment-induced changes translate into functional alterations in epithelial adhesion.

Taken together, these findings indicate selective interactions between OE and LE and the bacterial strains investigated, rather than a generalized stimulation of probiotic growth. Importantly, the present experimental design does not allow these effects to be classified as prebiotic activity, nor can they be directly extrapolated to modulation of the complex gut microbiota. They should instead be regarded as preliminary evidence of strain-specific interactions between *Citrus*-derived plant complexes and selected beneficial bacteria.

When considered together with the epithelial findings, these results indicate that standardized plant complexes recovered from *Citrus* processing by-products retain measurable biological activity after simulated gastrointestinal digestion and affect distinct epithelial and selected bacterial endpoints in vitro. Future studies employing more complex microbial communities, ex vivo fermentation systems, and host–microbiota co-culture models will be required to establish the biological relevance of these interactions in more physiologically representative settings.

## 5. Conclusions

The present study provides additional in vitro evidence of the biological activity of standardized food-grade *Citrus* pomace-derived plant complexes in intestinal epithelial and selected bacterial models. Following simulated gastrointestinal digestion, OE and LE digestates retained their biological activity, promoting epithelial wound closure while modulating the extracellular levels of oxidative stress-related proteins and pro-inflammatory cytokines in differentiated Caco-2 monolayers without affecting cell viability. These findings complement the previously reported effects on epithelial barrier-associated endpoints and indicate that the digestates can modulate additional epithelial responses under the in vitro experimental conditions employed.

Beyond their effects on intestinal epithelial cells, OE and LE, both before and after simulated gastrointestinal digestion, differentially modulated the growth and cell-surface hydrophobicity of selected probiotic strains in a strain-dependent manner, providing preliminary evidence of interactions between these *Citrus*-derived plant complexes and selected beneficial microorganisms. Although these observations were obtained using a simplified in vitro bacterial model, they should be interpreted as evidence of strain-specific effects on bacterial growth and surface physicochemical properties, rather than as a demonstration of probiotic or prebiotic activity, improved intestinal colonization, or modulation of the gut microbiota, and warrant further investigation in more complex microbial systems.

Overall, these findings provide a rationale for further investigating standardized *Citrus* pomace-derived plant complexes as sustainable food-grade ingredients potentially relevant to gut health applications. Nevertheless, the present findings were obtained using in vitro experimental models, and further studies employing complex microbial communities, integrated host–microbiota models, and in vivo approaches, will be required to elucidate the underlying molecular mechanisms and determine whether the effects observed here translate into biologically relevant outcomes under physiologically representative conditions and, ultimately, into nutraceutical efficacy.

## Data Availability

The original contributions presented in this study are included in the article. Further inquiries can be directed to the corresponding author.

## Abbreviations

The following abbreviations are used in this manuscript:

ANOVA	Analysis of variance
CTR−	Negative control
CTR+	Positive control
DIGs	Digestates
DMEM	Dulbecco’s Modified Eagle’s Medium
DMSO	Dimethyl sulfoxide
ELISA	Enzyme-linked immunosorbent assay
FBS	Fetal bovine serum
GAE	Gallic acid equivalents
IBD	Inflammatory bowel disease
IL-1β	Interleukin-1 beta
IL-6	Interleukin-6
IL-8	Interleukin-8
LAB	Lactic acid bacteria
LE	Lemon pomace extract
LPS	Lipopolysaccharide
MAS	Microbial adhesion to solvents
MEM-NEAA	Minimum Essential Medium non-essential amino acids
MRS	de Man, Rogosa and Sharpe
MTT	3-(4,5-Dimethylthiazol-2-yl)-2,5-diphenyltetrazolium bromide
Nrf2	Nuclear factor erythroid 2-related factor 2
OD600	Optical density at 600 nm
OE	Orange pomace extract
PBS	Phosphate-buffered saline
PET	Polyethylene terephthalate
ROS	Reactive oxygen species
SD	Standard deviation
SOD2	Superoxide dismutase 2
TEER	Transepithelial electrical resistance
TNF-α	Tumor necrosis factor alpha
